# Effect of *Lentinus sajor-caju* on the chemical composition and antioxidant activity of highland barley straw under solid-state fermentation

**DOI:** 10.3389/fmicb.2024.1365254

**Published:** 2024-05-22

**Authors:** Yuqiong Wang, Yangci Liao, Changlong Gou, Hang Zhang, Liming Chen, Yuhong Bao

**Affiliations:** ^1^College of Animal Science and Technology, Inner Mongolia Minzu University, Tongliao, Inner Mongolia, China; ^2^Institute of Pratacultural, Tibet Academy of Agricultural and Animal Husbandry Sciences, Lhasa, Tibet, China

**Keywords:** solid-state fermentation, antioxidant activity, highland barley straw, LC–MS, *Lentinus sajor-caju*

## Abstract

**Introduction:**

The efficient utilization of straw resources as animal feed has gained considerable attention. The objective of this study was to evaluate whether *Lentinus sajor-caju* treatment alters the chemical composition and antioxidant activity of highland barley straw and enhances its functional value as a ruminant feed.

**Methods:**

The chemical composition, antioxidant capacity, and metabolomic profile of highland barley straw were determined after 21 days of solid-state fermentation with *L. sajor-caju* at 25°C. The differential metabolites between fermented and unfermented highland barley straw were identified by LC–MS and the relationship between the identified metabolites and antioxidant capacity was elucidated.

**Results:**

The results showed that, compared with untreated highland barley straw, the crude protein and ether extract contents were higher (51.55 and 76.43%, respectively) in highland barley straw after 21 days of incubation with *L. sajor-caju*, whereas the hemicellulose, cellulose, and acid detergent lignin contents were lower (2.48, 25.08, and 45%, respectively). The total antioxidant capacity was significantly higher in *L. sajor-caju*-treated than in untreated highland barley straw. In total, 600 differential metabolites (301 upregulated and 299 downregulated) were identified between *L. sajor-caju*-fermented and unfermented highland barley straw. Correlation analysis results showed that Fe^2+^ scavenging and total phenolic content were strongly correlated with total antioxidant capacity. Meanwhile, the differential flavonoid metabolites between fermented and unfermented highland barley straw were primarily associated with antioxidant activity, with kaempferol 3-xylosylglucoside, isoginkgetin, and rhoifolin being the most representative.

**Conclusion:**

Thus, this study demonstrates that *L. sajor-caju* could enhance the functional value of highland barley straw, showing the potential of *L. sajor-caju* for improving the utilization of agricultural straws in ruminants.

## 1 Introduction

China produces a variety of crop residues, including straw, vine, grain, and fruit byproducts, which are generally used to supplement roughage in animal feed. Highland barley (HB) straw is a byproduct of the local staple food crop in the Qinghai-Tibet region of China. Improving the nutritional value of HB straw has the potential to enhance the utilization ratios of ruminants and alleviate feed resource shortages. However, these substances contain lignin, which is the main factor responsible for low direct utilization ratios of animal feedstock (Datsomor et al., 2022). Lignin, a widely abundant polyphenolic resource with potential antioxidant activities, exhibits very poor biodegradability (Figueiredo et al., 2018). Therefore, finding means of enhancing the utilization and antioxidant activity of lignin is important for promoting high-efficiency straw feed utilization. Arora et al. (2011) reported that four white rot fungi were able to degrade lignin and enhance different antioxidant properties of wheat straw up to 30 days under solid state conditions, which were verified had a positive correlation total phenolic content (TPC) with lignin loss. The scanning electron microscopy (SEM) found that the lignocellulose structure showed fragmentation under solid state conditions of wheat bran by white rot fungi for 12 days. The substrate increased the levels of active components that regulate the antioxidant in poultry (Wang et al., 2017). Thereout, white rot fungi can break the covalent linkages of lignin (Cui et al., 2021), leading to the formation of low-molecular-weight phenolic compounds with increased antioxidant activity. Phenolic compounds have been shown to reduce the levels of lipid peroxyl radicals, inhibit lipid peroxidation, and scavenge free radicals present in the body (Liu et al., 2020, 2022). Antioxidants enhance cellular immunity and repair, thus maintaining the function and structure of key immune cells (Sharma et al., 2010), and they are very important for the immune defense functions and health of animals in animal production. Thus, a lack of antioxidants can lead to a decline in immunity, resulting in an increase in the incidence of animal diseases and a reduction in animal performance.

*Lentinus sajor-caju*, a white rot fungus belonging to the phylum Basidiomycota, is rich in amino acids, vitamins, minerals, and other nutrients, except fats. This species has significant amounts of phenolic compounds such as phenols and flavonoids, which are known to possess antitumor, antioxidant, anti-inflammatory, and antimicrobial properties, and hence has high nutritional and medicinal value (Boonsong et al., 2016; Finimundy et al., 2018). In addition, *L. sajor-caju* are the better degraders of lignin under different substrate conditions. Lignin of tea leaf waste was decreased by 35.63% and improved nutritional value under solid state fermentation for 56 days with *L. sajor-caju* (Hossain et al., 2016)*. Lentinus sajor-caju* caused the higher lignin loss (29.92%) compared to other white rot fungi in beech sapwood (*Fagus sylvatica*) lumber (Bari et al., 2021). Lignin contents was decreased by 72.33% in water-hyacinth by *L. sajor-caju* after 56 days of fermentation. Thus, it is speculated that *L. sajor-caju* mycelium propagation or its lignin degradation can produce polyphenols and improve the antioxidant capacity.

Despite the continued search for nutritional value in agricultural byproducts using *L. sajor-caju*-based fermentation, antioxidant production in the fermentation substrate is limited. Whether the *L. sajor-caju*-mediated fermentation of agricultural waste can enhance the antioxidant activity of fermentation substrates merits further consideration. It is therefore of interest to identify the secondary metabolites and nutritional components responsible for the antioxidant properties of *L. sajor-caju*. The aim of this study was to determine the chemical composition, antioxidant activity, and metabolomic profile of HB straw after treatment with *L. sajor-caju*. Our results may help guiding the development of functional feed and contribute to enhancing the immune defense of animals during animal production.

## 2 Materials and methods

### 2.1 Organisms and substrate

*Lentinus sajor-caju* (Fr.) Fr. (CGMCC 5.592) was procured from the China General Microbiological Culture Collection Center (CGMCC) in Beijing, China. The fungus was cultured on yeast extract glucose agar (YGA) slants and stored at 4°C. Highland barley straw was collected from Mozhu Gongka County, Tibet, China (29°50′ N, 91°45′ E).

### 2.2 Substrate preparation and inoculation

The HB straw was chopped into 2–3 cm-long pieces and distilled water was added until 65% moisture was reached. A total of 200 g of wet HB straw was placed in an autoclavable plastic bag and autoclaved at 121°C for 30 min, and then allowed to cool to room temperature. The isolated fungus was incubated on YGA plates at 25°C for 7 days. The five disks (0.5 cm^2^) of active mycelium from an YGA plate were transferred aseptically into wet HB straw for 21 days of solid-state fermentation in a climatic chamber at 25 ± 0.5°C. In the control group (CK), no inoculation was performed. Six replicates were used throughout the experiment.

### 2.3 Chemical composition and antioxidant potential

#### 2.3.1 Chemical analysis

The co-fermentation samples were dried to constant weight at 60°C. The crude protein (CP) content was determined according to method 990.06 of AOAC (2000), with a conversion factor of 6.25. Ethyl ether extract (EE) contents were determined using AOAC method 2003.05. The ash content was calculated by ashing at 550°C in a muffle furnace for 3 h. Neutral detergent fiber (NDF), acid detergent fiber (ADF), cellulose (CL), hemicellulose (HC), and acid detergent lignin (ADL) contents were assessed as described by Goering and Soest (1970) and Van Soest et al. (1991), with slight modifications. Samples (0.5–1 g) were placed in polyester mesh bags (Ankom F57), sealed, and, along with 2,000 mL of neutral detergent, placed in a semi-automatic fiber analyzer (ANKOM 200i) at 100°C for 60 min. The bags were then washed to neutral with distilled water, dried, and weighed. The dried residue was considered to be NDF. The remaining samples along with 2,000 mL of acid detergent were placed in the semi-automatic fiber analyzer at 100°C for 60 min. Subsequently, the bags were again washed to neutral with distilled water, dried, and weighed. The dried residue represented ADF, whereas the loss represented HC. The dried residue was soaked in 72% (*v*/*v*) H_2_SO_4_ and left to stand at 25°C for 2 h. Thereafter, the bags were washed to neutral with distilled water, dried, and weighed, and the loss represented CL. The remaining samples were kept at 550°C for 3 h in a tared crucible and reweighed, and the loss was considered to represent the ADL.

#### 2.3.2 Extraction

Fresh samples were randomly divided into two samples of 80 g, air-dried in an oven at 40°C, and mixed with extraction medium at a ratio of 1:10 (*w*/*v*). The samples were extracted with methyl alcohol at room temperature at 150 rpm for 24 h. The residue was then re-extracted as described above. Each sample was filtered using filter paper (Whatman No.1) and the filtrate was used for the determination of antioxidant activity.

#### 2.3.3 Quantitative assay for free radical scavenging activity (DPPH)

The scavenging activity for DPPH free radicals was measured according to Arora and Chandra (2010). A 1-mL aliquot of 0.1 mM solution of DPPH in ethanol and 0.5 mL of extract were mixed and the mixture was shaken vigorously and allowed to reach a steady state at 37°C for 30 min. The scavenging activity was calculated as follows:

$$\%\mathrm{scavenging}\;\mathrm{rate}=\left[1-\left(A1-A2\right)/A0\right]\times 100$$

where *A*0 is the absorbance of the control, *A*1 is the absorbance of the extract, and *A*2 is the absorbance without DPPH.

#### 2.3.4 Determination of antioxidant activity *via* the measurement of reducing power

The reducing power of the extracts was determined according to Chang et al. (2002). The extract (0.5 mL) was mixed with 0.1 mL of potassium ferricyanide (10 g/L), followed by incubation at 50°C for 30 min. The reaction mixture was supplemented with 0.1 mL of trichloroacetic acid (10 g/L) and FeCl_3_ (1 g/L) for 20 min. Absorbance was measured at 700 nm. The higher the absorbance of the reaction mixture, the higher the reducing power of the sample.

#### 2.3.5 Determination of antioxidant activity by ferric reducing antioxidant power assay

The FRAP assay was carried out according to Othman et al. (2007). The FRAP reagent was prepared by mixing 300 mM acetate buffer (pH 3.6), 10 mM TPZE, and 20 mM ferric chloride in a ratio of 10:1:1 (*v*/*v*). The extract (0.5 mL) and distilled water (1 mL) were added to the reaction mixture, followed by incubation for 10 min. Absorbance was measured at 593 nm. The antioxidant potential of the sample was compared with that of a 0.5-mL stock solution of 1 mg/mL FeSO_4_.

#### 2.3.6 Determination of ferrous ion scavenging (metal chelating) activity

The ferrous ion chelating activity of the extracts was measured according to Zhao et al. (2006). The reaction mixture was incubated with 0.5 mL of extract, 1.6 mL of deionized water, 0.05 mL of FeCl_2_ (2 mM), and 0.1 mL of ferrozine (5 mM) at 40°C for 10 min, after which the absorbance at 562 nm was measured. The chelating activity was calculated as:

$$\mathrm{Chelating}\;\mathrm{rate}=\left[1-\left(A1-A2\right)/A0\right]\times 10$$

where *A*0 is the absorbance of the control, *A*1 is the absorbance of the extract, and *A*2 is the absorbance without FeCl_2_.

#### 2.3.7 Determination of total phenolic content

Total phenolic content was determined as described by Singleton et al. (1999). The extract (0.5 mL) was mixed with Folin–Ciocalteu reagent (0.2 mL) and allowed to stand at room temperature for 10 min. The reaction mixture was supplemented with 0.6 mL of sodium carbonate (20% *w*/*v*) and the absorbance at 765 nm was measured. Gallic acid was used as a standard.

### 2.4 Metabolomic profiling and multivariate analysis

For the internal standard, lyophilized powdered samples (50 ± 0.1 mg) were extracted with 1,000 μL of extract solution (methanol: H_2_O = 3:1 [*v*/*v*]) supplemented with an internal standard mixture. The samples were sonicated in an ultrasonic bath (SB25-12D, Xinzhi, Ningbo, China) for 5 min and centrifuged at 12,000 rpm at 4°C for 15 min. The supernatant was collected and analyzed by ultra-high-performance liquid chromatography coupled with high-resolution mass spectrometry (LC–MS). Metabolomic analysis was performed using an UHPLC (Thermo Fisher Scientific, Waltham, MA, United States). Chromatographic separation was performed on an ACQUITY UPLC BEH Amide column (100 mm × 2.1 mm, 1.7 μm particle size; Waters, United States) maintained at 4°C. The mobile phase consisted of 25 mM ammonium acetate and 25 mM ammonium hydroxide (eluent A) and acetonitrile (eluent B) and the flow rate was set at 0.5 mL/min. The gradient elution program was as follows: 0.5 min, 95% B; 0.5–7 min, 95–65% B; 7–8 min, 65–40% B; 9 min, 40% B; 9–9.1 min, 40–95% B; and 12 min, 95% B. The sample injection volume was 2 μL and the temperature of the autosampler was maintained at 4°C. For MS analysis, the parameters for positive ion mode were set as follows: ion transfer capillary temperature, 350°C; collision energy: 10/30/60 in normalized collision energy mode; needle voltage, +3,600 V; sheath gas pressure, 30 arbitrary units; auxiliary gas pressure, 25 arbitrary units. For negative ion mode, the differing parameters were 300°C for the capillary temperature and − 3,200 V for the needle voltage.

### 2.5 Statistical analysis

Data were analyzed by one-way ANOVA using SAS 2008. Means are presented as least-square means ± standard error of the mean (SEM). Correlations between ADL content loss and potential antioxidant parameters (Spearman rank correlation coefficients) were considered strongly positive and significant at *r* > 0.5 and *p* < 0.05. Similarly, correlations were considered strongly negative and significant when *r* > −0.5 and *p* < 0.05.

Missing values in the metabolomics dataset were replaced with minimal values. Differential metabolomic analysis was performed on log_2_-transformed data. A Student’s *t*-test *p* value <0.05, an adjusted Fold Change (FC) >1.5 or < 0.65, and an OPLS-DA model Variable Importance in Projection (VIP) value >1 were used as the cut-off criteria.

## 3 Results

### 3.1 Chemical composition analysis

The nutrient composition (dry matter basis) of HB straw during solid-state fermentation by *L. sajor-caju* is shown in Table 1. Compared with untreated HB straw, the CP and EE contents were significantly higher (both *p* < 0.001) in HB straw after 21 days of inoculation with *L. sajor-caju*, whereas the NDF, ADF, HC, CL, and ADL contents were significantly lower (all *p* < 0.001). The content of CP increased by 51.55% and those of HC, CL, and ADL were reduced by 22.48, 25.08, and 45% with *L. sajor-caju* treatment compared with the control condition, respectively.

**Table 1:** Nutritional properties (g/100 g dry matter) of highland barley straw after its solid-state fermentation by *Lentinus sajor-caju*.

Item	CP	EE	NDF	ADF	HC	CL	ADL
CK	4.52^B^	1.40^B^	64.55^A^	39.99^A^	26.51^A^	20.85^A^	19.02^A^
LS	6.85^A^	2.47^A^	48.27^B^	31.54^B^	20.55^B^	15.62^B^	10.38^B^
SEM	0.3166	0.1459	0.726	0.845	1.223	1.035	0.587
*p* value	<0.001	<0.001	<0.001	<0.001	<0.001	<0.001	<0.001

^A,B^Within a row, means with different superscript letters differ significantly (*p* < 0.001). CK, Control group; LS, *L. sajor-caju* fermentation group; SEM, Standard error of the mean; CP, Crude protein; EE, Ether extract; NDF, Neutral detergent fiber; ADF, Acid detergent fiber; HC, Hemicellulose; CL, Cellulose; and ADL, Acid detergent lignin.

### 3.2 Antioxidant activity of the extracts

The antioxidant properties of HB straw incubated with *L. sajor-caju* are shown in Table 2. The antioxidant activity was significantly increased (*p* < 0.001) in HB straw incubated with *L. sajor-caju* for 21 days relative to that in untreated substrate. The TPC, Fe^2+^ scavenging activity, DPPH, FRAP, and reducing power were 182.12, 73.46, 63.66, 56.86, and 54.10% higher in fermented HB than in unfermented HB, respectively. These results indicated that there were significant differences in antioxidant activities between the two groups.

**Table 2:** Antioxidant properties of highland barley straw incubated with *Lentinus sajor-caju*.

Item	DPPH assay (%)	Reducing power (%)	FRAP assay (%)	Fe^2+^ scavenging (%)	TPC (mg/g)
CK	31.95^B^	0.61^B^	26.91^B^	10.89^B^	3.02^B^
LS	52.29^A^	0.94^A^	46.21^A^	18.89^A^	8.52^A^
SEM	0.67	0.04	0.74	0.55	0.28
*p* value	<0.001	<0.001	<0.001	<0.001	<0.001

^A,B^ Within a row, means with different superscript letters differ significantly (*p* < 0.001). CK, Control group; LS, *L. sajor-caju* fermentation group; SEM, Standard error of the mean; DPPH, 2,2-diphenyl-1-picrylhydrazyl; FRAP, Ferric reducing antioxidant power; TPC, Total phenolic content.

### 3.3 Metabolite differences and classification

#### 3.3.1 Differential metabolite analysis

**Figure 1:**
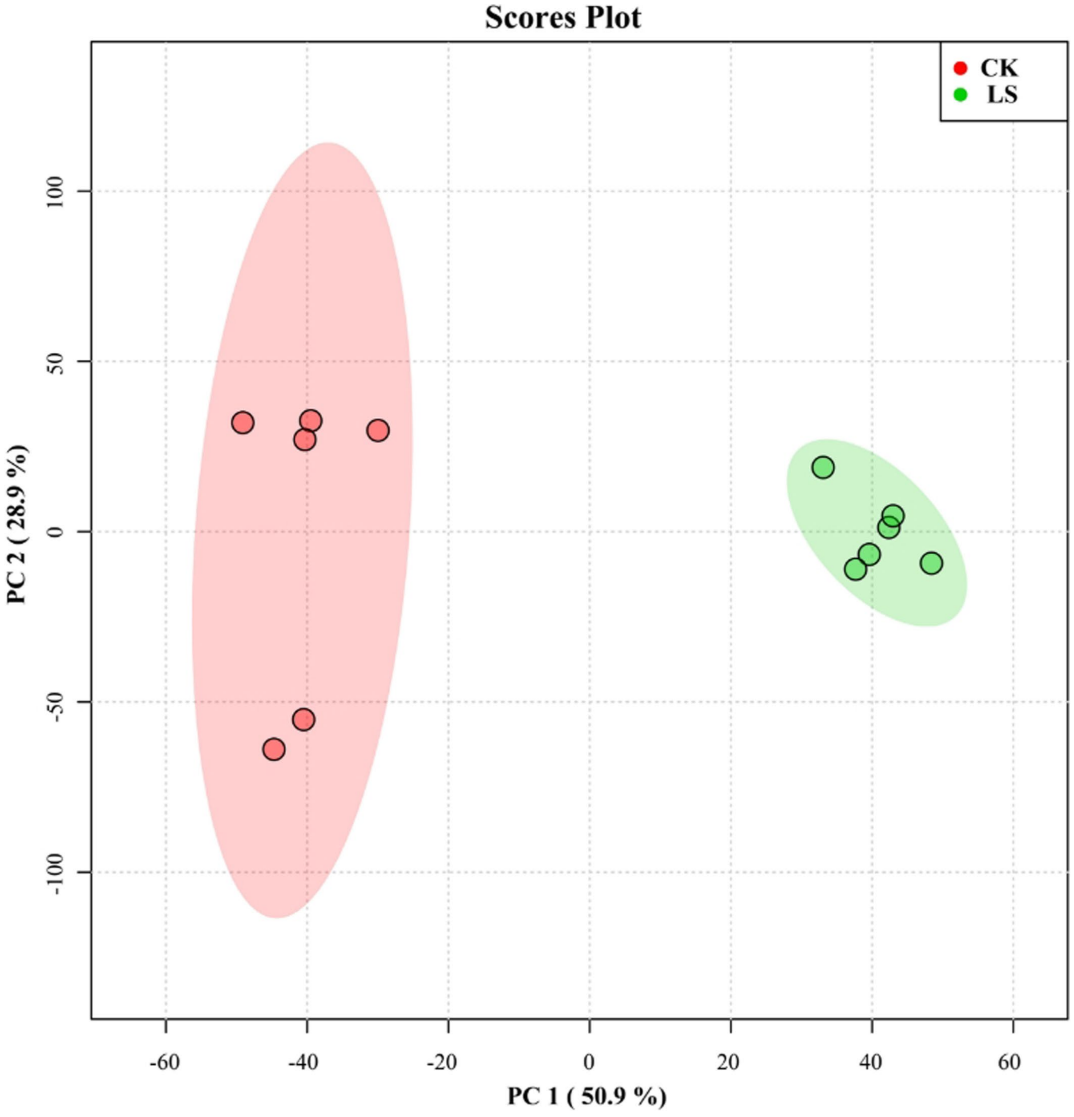
Principal component analysis (PCA) score plot for the control (CK) group and the *Lentinus sajor-caju* fermentation (LS) group.

Studies on extracellular *L. sajor-caju* metabolites are relatively scarce. Principal component analysis (PCA) (Figure 1) showed that the *L. sajor-caju* samples clustered together distant from those of the control. In the PCA score plot, two principal components (PC1 and PC2) were extracted and explained 50.9 and 28.9% of the metabolite variation, respectively. The results showed that the two treatment groups were clearly separated and that all six of the biological replicates of each group were tightly clustered, indicating that the experiment was reproducible and reliable.

#### 3.3.2 Differential metabolite screening, functional analysis, and annotation

**Figure 2:**
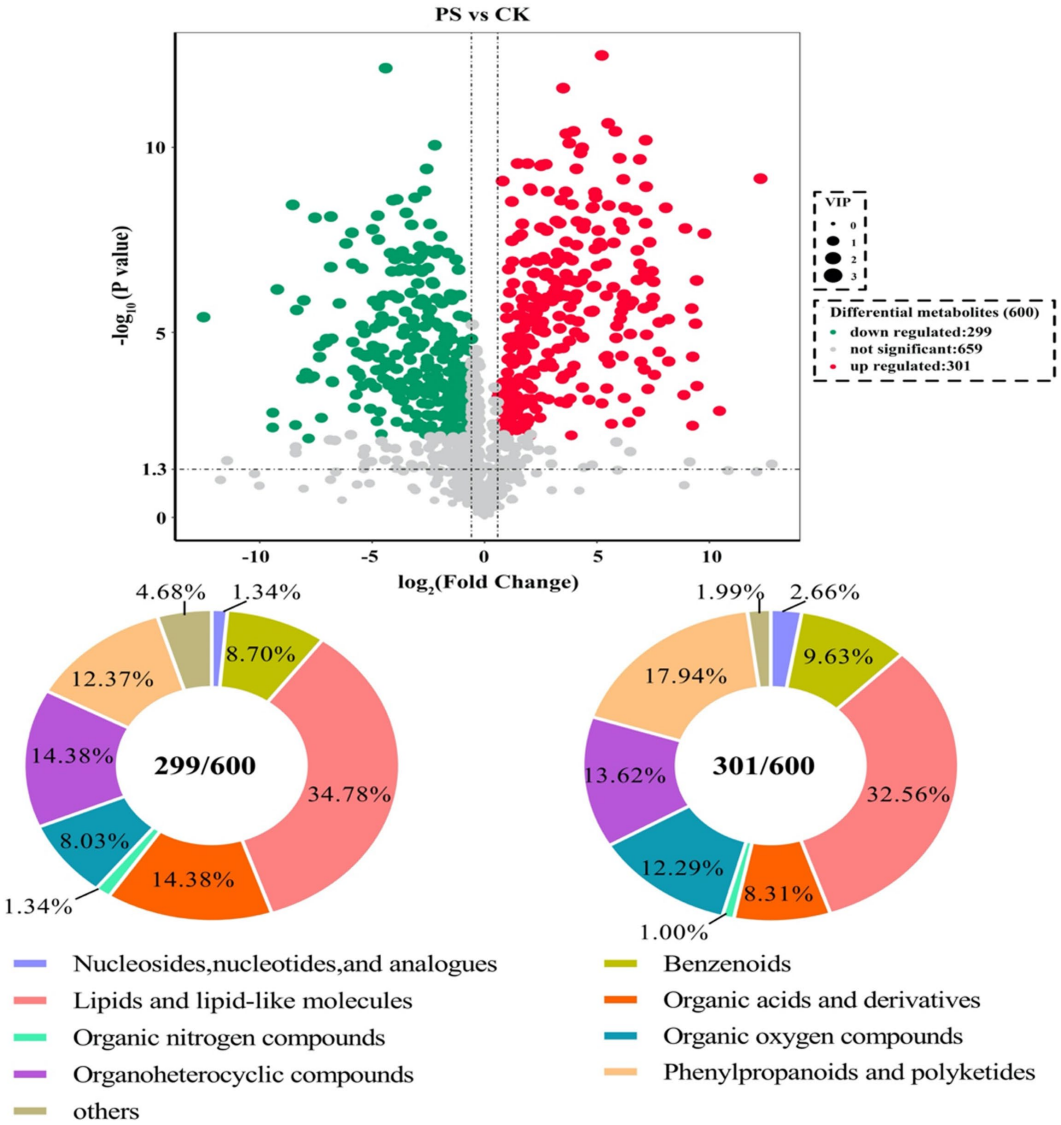
Analysis of the identified metabolites. Volcano plot showing differentially abundant metabolites (Fold Change <0.67 or > 1.5, *p* value < 0.05) between the control (CK) group and the *Lentinus sajor-caju* fermentation (LS) group. The types of compounds that were differentially abundant are shown in the pie charts.

The metabolites showing significant differential abundance between the *L. sajor-caju* treatment group and the control group are shown in the volcano plot in Figure 2. Based on an in-house metabolite database, qualitative and quantitative mass spectrometric analyses were conducted on the metabolites in the samples. A total of 600 compounds showed significant differential abundance between the two groups. Compared with the CK group, 301 compounds were significantly upregulated and 299 were significantly downregulated in the *L. sajor-caju*-treatment group. Of the 600 differentially abundant metabolites, 202 were lipids or lipid-like molecules (33.67%), 91 were phenylpropanoids and polyketides (15.17%), 84 were organoheterocyclic compounds (14%), 68 were organic acids and their derivatives (11.33%), 61 were organic oxygen compounds (10.17%), 55 were benzenoids (9.17%), 12 were nucleosides and their derivatives (2%), 7 were organic nitrogen compounds (1.17%), 5 were alkaloids and their derivatives (0.83%), 3 were organosulfur compounds (0.5%), and 12 were other metabolites (2%).

**Figure 3:**
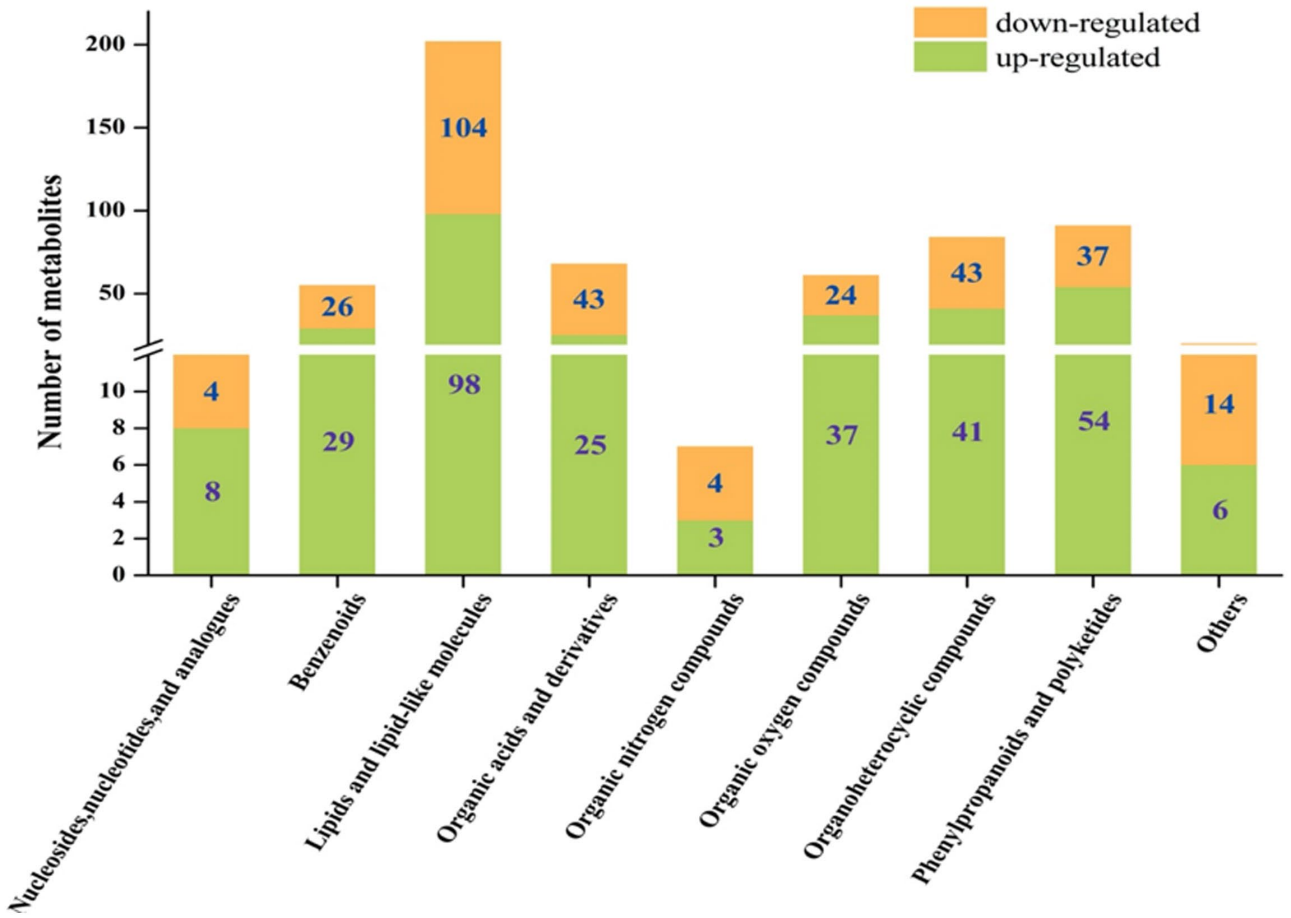
Column chart of the fold changes of the different superclass classifications.

Despite the commonalities between the upregulated and downregulated metabolites, we found that there were some differences between the fermented and unfermented HB straw. In particular, among the upregulated metabolites, benzenoids, phenylpropanoids and polyketides, organic oxygen compounds, and nucleosides and their analogs were 1.12-, 1.39-, 1.54-, and 2-fold more abundant in the *L. sajor-caju* group than in the CK group, whereas lipids and lipid-like molecules, organoheterocyclic compounds, organic acids and their derivatives, and organic nitrogen compounds were 1.06-, 1.05-, 1.72-, and 1.33-fold lower with *L. sajor-caju* treatment than under the control condition (Figure 3).

#### 3.3.3 Phenolic compounds composition in HB straw fermented by *Lentinus sajor-caju*

It is well known that edible fungi are rich in phenolic compounds. In the present study, we investigated the composition and contents of phenolic compounds and sought to elucidate the potential chemical basis underlying the differences in antioxidant activity between unfermented and *L. sajor-caju*-fermented HB straw.

**Figure 4:**
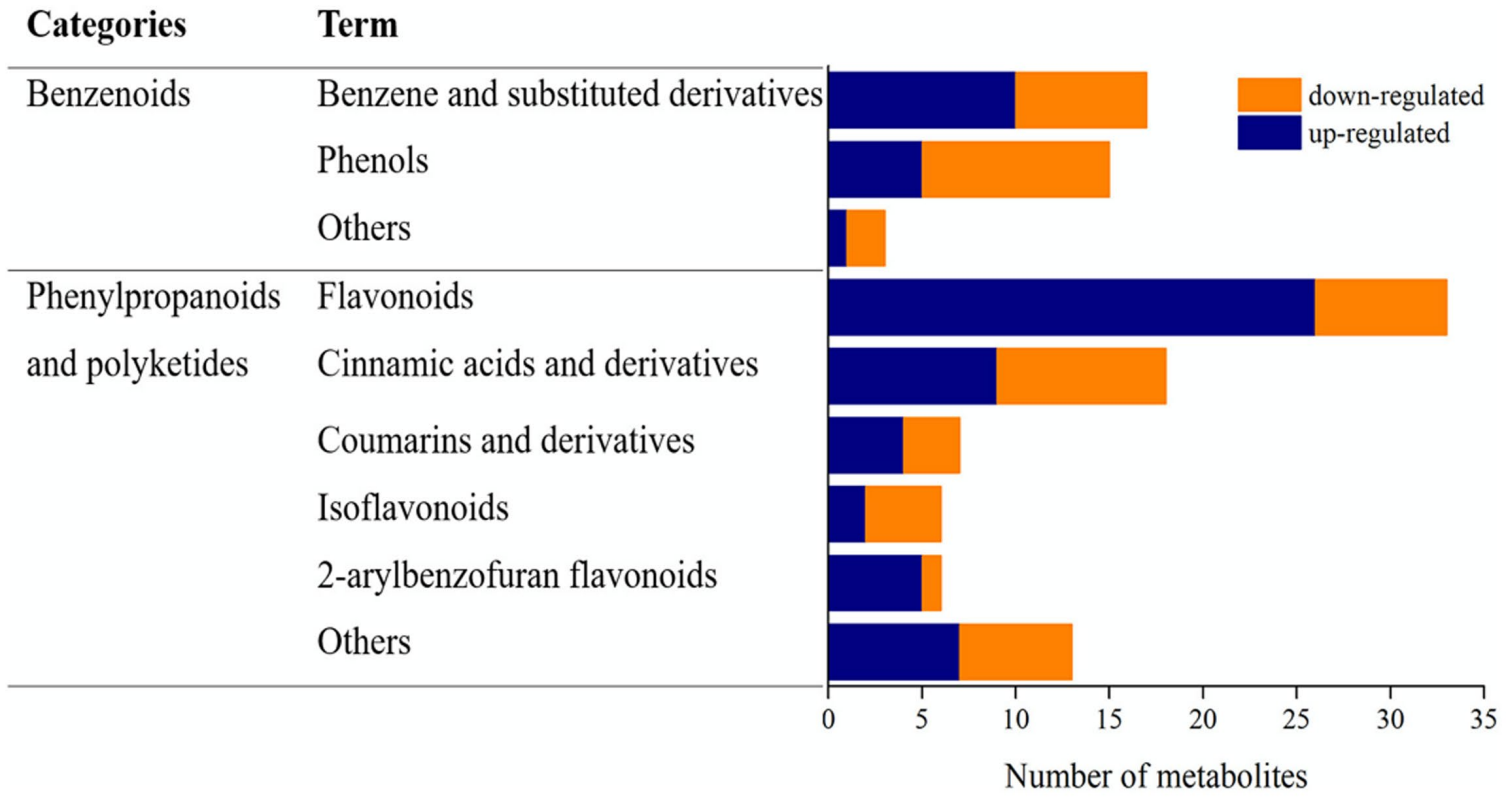
Benzenoids and phenylpropanoids/polyketides among the upregulated and downregulated metabolites in unfermented and *Lentinus sajor-caju*-fermented highland barley straw.

The phenolic compounds among the metabolites were categorized as benzenoids or phenylpropanoids and polyketides (Figure 4). Among the benzenoids, 17 were classified as benzene and substituted derivatives, 15 were phenols, and three belonged to other groups. Among the phenylpropanoids and polyketides, 33 were flavonoids, 18 were cinnamic acids and their derivatives, seven were coumarins and their derivatives, six were isoflavonoids, six were 2-arylbenzofuran flavonoids, and 12 were classified as “others.” Benzene and substituted derivatives and phenols were the main phenolic compounds in the benzenoid category. Flavonoid metabolites were predominant among the phenolic compounds in the phenylpropanoid and polyketide category; 26 flavonoids were upregulated in the *L. sajor-caju* treatment group compared with that in the CK group.

**Figure 5:**
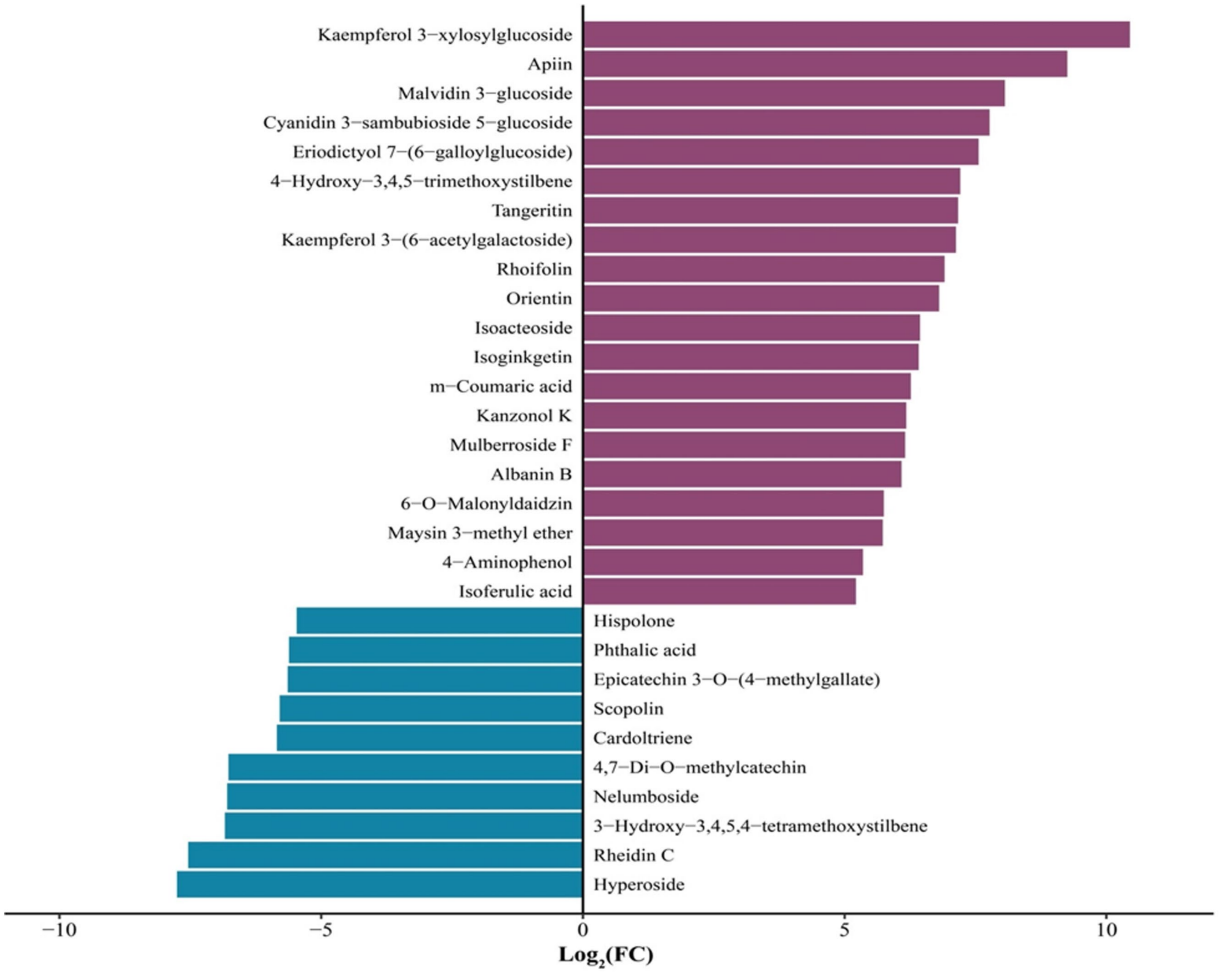
The 30 phenolic metabolites that displayed the greatest variation (log_2_ FC > 5) between unfermented and *Lentinus sajor-caju*-fermented highland barley straw.

The 30 phenolic compound-related metabolites with the highest FC variation between unfermented and *L. sajor-caju*-fermented HB straw are shown in Figure 5. During the *L. sajor-caju*-mediated fermentation of HB straw, the content of flavonoid metabolites was significantly increased compared with that in unfermented HB straw, whereas that of phenol-based metabolites was significantly decreased (log_2_ FC > 1). Among the 15 flavonoid metabolites, 12 were upregulated (log_2_ FC ranging from +5.73 to +10.45) and 3 were downregulated (log_2_ FC: −6.77 to −7.76) in the *L. sajor-caju* treatment group compared with that in the CK group. Two phenol metabolites were also downregulated (log_2_ FC: −5.84 to −5.46) in fermented HB straw. The greatest magnitude of change among the metabolites was observed with kaempferol 3-xylosylglucoside (log_2_ FC > 10).

### 3.4 Analysis of the correlation of lignin and phenolic compounds with antioxidant activity

**Figure 6:**
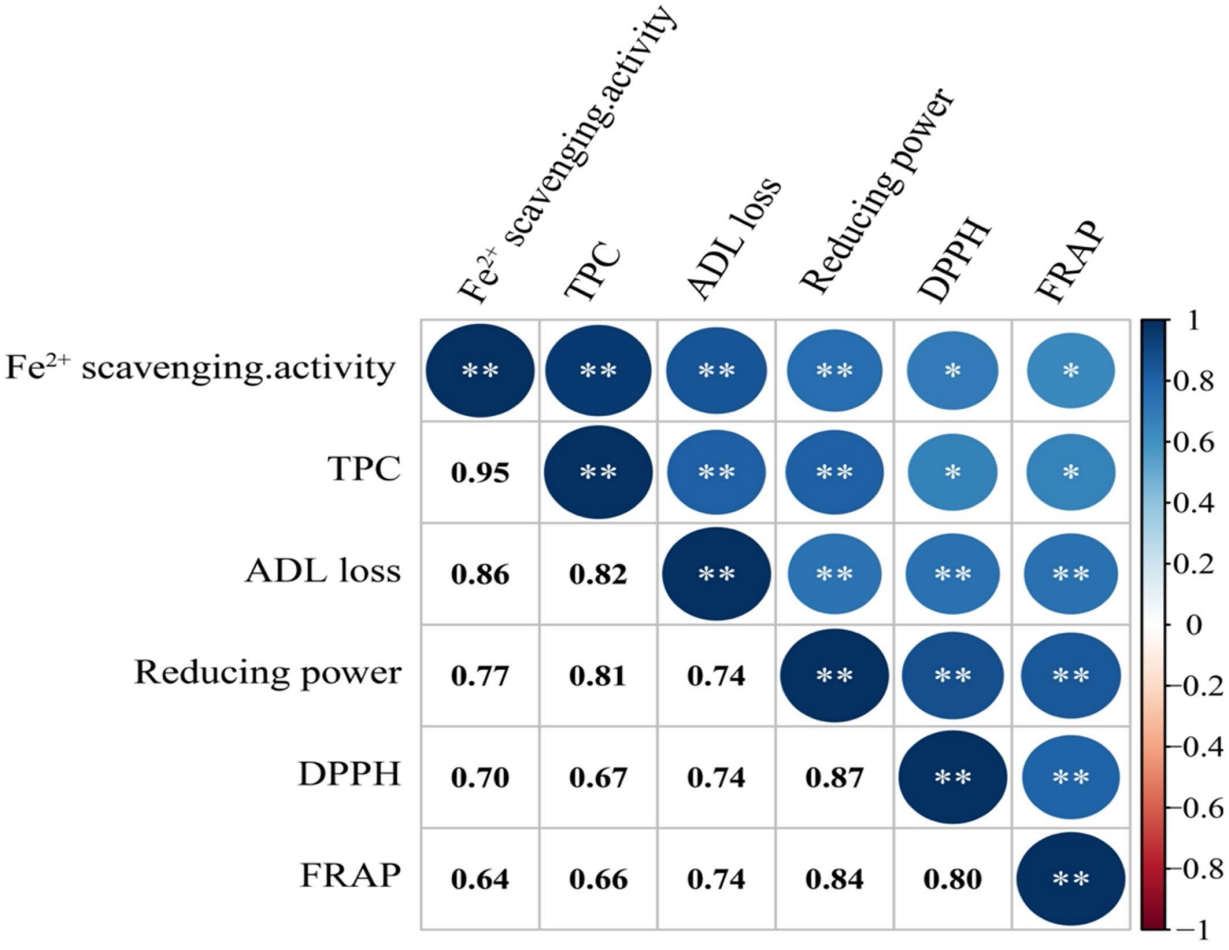
Spearman rank correlation coefficient (*r*) between the different parameters studied. DPPH, 2,2-diphenyl-1-picrylhydrazyl; FRAP, Ferric reducing antioxidant power; TPC, Total phenolic content; and ADL, Acid detergent lignin.

The results of the analysis of the putative correlations between lignin contents and antioxidant properties are shown in Figure 6. A positive correlation was found between the loss of lignin contents and Fe^2+^ scavenging and between the loss of lignin contents and TPC (*r* = 0.86 and 0.82, respectively), indicative of an increase in phenolic contents during lignin breakdown. Correlation analysis further indicated that Fe^2+^ scavenging and TPC were the major contributors to the antioxidant activity of *L. sajor-caju*-fermented HB straw.

**Figure 7:**
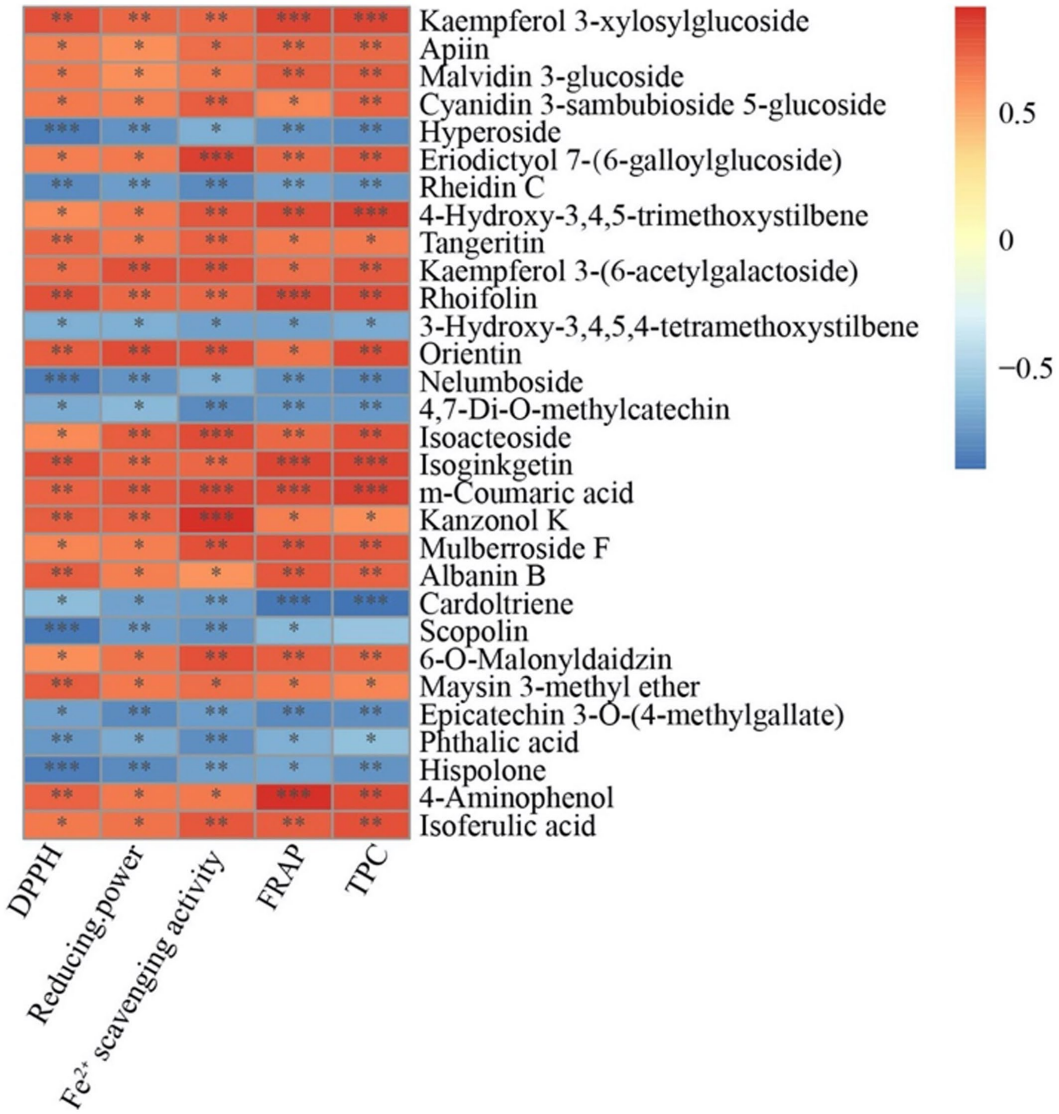
Correlation analysis between the top 30 phenolic metabolites and antioxidant properties. The right side shows the names of the top 30 phenolic metabolites and the bottom indicates DPPH, reducing power, Fe^2+^ scavenging, FRAP, and TPC. Each grid represents the correlation between the two attributes and different colors represent the sizes of the correlation coefficients between the attributes. ^*^p < 0.05, ^**^p < 0.01, ^***^p < 0.001. DPPH, 2,2-diphenyl-1-picrylhydrazyl; FRAP, Ferric reducing antioxidant power; and TPC, Total phenolic content.

The results of the correlation analysis between the top 30 phenolic compound-derived metabolites and the TPC, Fe^2+^ scavenging activity, free radical scavenging activity, and FRAP are depicted in Figure 7. Hyperoside, rheidin C, 3′-hydroxy-3,4,5,4′-tetramethoxystilbene, nelumboside, 4′,7-di-*O*-methylcatechin, cardol triene, epicatechin 3-*O*-(4-methylgallate), phthalic acid, and hispolone were significantly negatively correlated with free radical scavenging activity, reducing power, FRAP, Fe^2+^ scavenging activity, and TPC. m-Coumaric acid, a cinnamic acid, was significantly (*p* < 0.001) and positively correlated with FRAP, Fe^2+^ scavenging activity, and TPC. The flavonoids kaempferol 3-glucoside, isoginkgetin, and rhoifolin were significantly (*p* < 0.01) and positively correlated with free radical scavenging activity, reducing power, FRAP, Fe^2+^ scavenging activity, and TPC.

## 4 Discussion

White rot fungi can not only enhance the degradation of lignin but also improve the nutritional value of lignocellulosic biomass (Andrade et al., 2021). Basidiomycetes comprise a group known as white rot fungi that have been widely studied for their exceptional ligninolytic properties (Bellettini et al., 2019). We previously found that *L. sajor-caju* was superior at selectivity degrading lignin compared with five white rot fungi (Wang et al., 2023). In addition, the growth of fungal mycelia on HB straw was shown to contribute to the total protein content of feed. The finding was in accordance with the reports of Mahmoud (2006) and Shamim et al. (2017) lignin, cellulose and hemicelluloses were decreased by 19.0, 28.7, 31.0, and 72.33%, 37.56, and 24.24% of the water hyacinth after 49 and 56 days in solid state fermentation by *L. sajor-caju*. Cellulose degradation is a usually slightly lower with hemicellulose during solid state fermentation of lignocelluloses (Jahromi et al., 2011; Shamim et al., 2017). In this study, hemicellulose degradation was found lower was 22.48% compared to non-fermented. As reported earlier (Mahal et al., 2013), increased the CP content of jute plants and jute stick were converted into upgraded animal by solid state fermentation using *L. sajor-caju.* These observations demonstrated that white rot fungi can enhance the nutritional quality of fermentation substrates.

The antioxidant activity of putative antioxidants has been attributed to a variety of mechanisms, including the prevention of chain initiation, the binding of transition metal ion catalysts, the decomposition of peroxides, the prevention of continued hydrogen abstraction, reductive capacity, and radical scavenging (Gülçin et al., 2003). Owing to an easily detectable color reaction, DPPH is commonly used as a substrate for evaluating the antioxidative activity of antioxidants. Because ferrozine can quantitatively form complexes with Fe^2+^, the antioxidant activity of a fermentation substrate can be evaluated through the inhibition of the formation of red ferrous and ferric complexes. In the present study, we found that the fermentation substrate had strong antioxidant activity following 21 days of incubation with *L. sajor-caju*. In addition, the total phenolic content and the antioxidant activity of the substrate were significantly enhanced under *L. sajor-caju* treatment, which is in line with that reported in other studies (Elhusseiny et al., 2021). Antioxidants enhance immunity by maintaining the functional and structural integrity of key immune cells. Without adequate antioxidant reserves, the effectiveness of the immune response is reduced. Thus, the presence of antioxidants in animal feed is very useful for improving animal production performance. *Lentinus sajor-caju* produces a variety of oxidation-resistant components and degrades lignin, yielding polyphenols. Specifically, the antioxidant capacity of white rot fungi is also closely related to the pattern of lignin decomposition, the quality and quantity of small phenol units formed, their solubility, and differences in further transformation (Yang et al., 2010).

Of all the metabolites, the most highly represented superclass in fermented HB straw was lipids and lipid-like molecules, while organoheterocyclic compounds were among the top three represented superclasses; this was in accordance with that reported for fermented wheat straw, tobacco, and tea leaves. The contents of phenylpropanoids and polyketides in fermented wheat straw and tobacco leaves were lower than those detected in fermented HB straw in our study, whereas benzenoids displayed the opposite trend (Li et al., 2020; Zhu et al., 2022, 2024). Phenylpropanoids, polyketides, and benzenoids are metabolites associated with antioxidants such as phenols, flavonoids, and isoflavonoids. In summary, the antioxidant activity was affected by substrate materials and inoculants. The conclusion can be drawn that metabolites were abundant of antioxidant with *L. sajor-caju* fermented HB straw.

Phenolic compounds are well-known for their antioxidant capacity, which is due to their high redox potential. Flavonoids are a group of polyphenolic compounds widely found in fruits, mushrooms, vegetables, and other food crops. The antioxidant activity of flavonoids, which represents their main biological activity, has been extensively studied. They act as free radical scavengers, stopping the chain reactions that occur during triglyceride oxidation (Barros et al., 2008), and have a variety of biological and pharmacological properties, including anticancer and anti-inflammatory effects (Ravishankar et al., 2013). Phenols, which are generally present in plant cell walls bound to polysaccharides, also exhibit antioxidant activity (Rao and Muralikrishna, 2004; Parker et al., 2005). It has been shown that the antioxidant potential of substrates subjected to fungal-mediated fermentation is closely linked to the TPC However, fungus-derived phenolic may not be the key contributors to the antioxidant activity of fermented substrates (Elhusseiny et al., 2021). A similar conclusion was reached in our study. The contents of phenol-based metabolites in HB straw were significantly decreased after 21 days of *L. sajor-caju* fermentation, despite the antioxidant activity of the *L. sajor-caju* fermentation substrate being strong. This agrees with a study by Matuszewska et al. (2018), in which it was reported that the metabolite fraction with the lowest phenol content isolated from *Cerrena unicolor* had the strongest antioxidant activity. However, Castorina et al. (2023) showed that the growth of *Pleurotus ostreatus* and *Ganoderma annularis* led to a significant reduction in the total phenolics contents in corncobs alone. The growth of basidiomycetes was associated with the consumption of phenolic compounds. The differences in the above finding may be related to the content of sugars and starches in the substrate. Basidiomycetes preferentially choose easily available carbon as energy sources, thus limiting lignin decomposition for a certain period of time. In the present study, 33 flavonoid and 15 phenolic compounds were found to be differentially abundant between fermented and unfermented HB straw. The 26 flavonoids that were upregulated were upregulated to a greater extent than the five upregulated phenols. This suggests that a greater abundance of flavonoid compounds may be correlated with an increase in antioxidant activities. Notably, however, whether mycelia can produce flavonoids remains controversial. Although flavonoid synthesis has been detected in mycelial cells, it remains unresolved whether fruiting bodies have a similar ability (Pukalski and Latowski, 2022). In this study, the total antioxidant capacity of the fermentation substrate was related to flavonoid abundance. HB straw was incubated with *L. sajor-caju* for 21 days during the mycelium production period; however, it is not clear whether the flavonoids among the extracellular metabolites originated from the mycelia, HB straw, or both. It has been proposed that, during lignin degradation, flavonoid polyphenolic compounds may be produced by the biotransformation of lignin degradation products under oxidative stress (Zhao et al., 2021).

Correlation analysis can help identify both metabolites with antioxidant properties and metabolic markers. In this study, kaempferol, m-coumaric acid, isoginkgetin, and rhoifolin were indentifying the main metabolic markers with antioxidant properties. Kaempferol is the dominant flavonol found in vegetables and fruits and is related to the cell wall matrix, which has strong antioxidant activity. *Aspergillus awamori* was reported to be able to biotransform phenolic glycosides of litchi pericarp into kaempferol, with significantly increased antioxidant activity (Lin et al., 2014). Huynh et al. (2016) showed that the presence of free form of the aglycone (kaempferol 3-glucoside) in cauliflower outer leaves by microbial fermentation. We obtained similar results in this study, namely, kaempferol was present in the form of glycosides, with kaempferol 3-xylosylglucoside being the most abundant metabolite detected. This suggested that kaempferol 3-xylosylglucoside plays an important antioxidant role. m-Coumaric acid (a cinnamic acid) is a phenolic compound found in plant extracts. In this study, m-coumaric acid was significantly and positively correlated with FRAP and TPC. Fe^2+^ scavenging and TPC were the major contributors to the antioxidant activity observed after 21 days of *L. sajor-caju* incubation. Therefore, m-coumaric acid plays a leading role in antioxidants with incubated with HB straw for 21 days by *L. sajor-caju*. The strong antioxidant properties of *L. sajor-caju*-fermented HB straw suggest that it has the potential for use in the production of healthy feed for ruminants, thus also providing additional health and economic benefits to consumers.

## 5 Conclusion

In this study, the significantly higher contents of CP and EE, and lower contents of lignocellulose during 21 days *L. sajor-caju* incubated HB straw. Correlation analysis further indicated that Fe^2+^ scavenging and TPC were the major contributors to the antioxidant activity of *L. sajor-caju*-fermented HB straw. In addition, LC–MS-based metabolomics identified 600 differential metabolites between unfermented and *L. sajor-caju*-fermented HB straw, 117 of which were phenolic compounds. Meanwhile, the identified differential flavonoid metabolites were primarily associated with antioxidant activity, with kaempferol 3-xylosylglucoside, m-coumaric acid, isoginkgetin, and rhoifolin being the most representative. These results indicate that *L. sajor-caju*-fermented HB straw has promise as a functional feed and may be a good natural source of antioxidants.

## Data availability statement

The original contributions presented in the study are included in the article/supplementary material, further inquiries can be directed to the corresponding author.

## Author contributions

YW: Data curation, Formal analysis, Writing – original draft, Writing – review & editing. YL: Data curation, Writing – review & editing. CG: Formal analysis, Investigation, Writing – review & editing. HZ: Investigation, Supervision, Writing – review & editing. LC: Writing – review & editing, Methodology. YB: Conceptualization, Supervision, Writing – review & editing.
